# Assessment of clinical probability scores for pulmonary embolism diagnosis during pregnancy and postpartum in women with a history of venous thromboembolism: a Highlow ancillary study

**DOI:** 10.1016/j.rpth.2025.103281

**Published:** 2025-12-05

**Authors:** Fanny Collange, Ingrid M. Bistervels, Andrea Buchmuller, Hanke M.G. Wiegers, Fionnuala Ní Áinle, Peter Verhamme, Anne F. Jacobsen, Anette T. Hansen, Marc A. Rodger, Maria T. DeSancho, Roman G. Shmakov, Luuk J.J. Scheres, Celine Chauleur, Saskia Middeldorp, Bernard Tardy, Highlow investigators, Barbara Debaveye, Kathelijne Peerlinck, Caroline P. Martens, Kristine Vanheule, Thomas Vanassche, Peter Verhamme, Marc A. Rodger, Alan Karovitch, Anette Tarp Hansen, Aiste Kloster, Jens Fuglsang, Andrea Buchmüller, Céline Chauleur, Cécile Duvillard, Nathalie Moulin, Tiphaine Raia-Barjat, Thomas Corsini, Suzanne Lima, Luc Bressollette, Francis Couturaud, Karine Lacut (deceased), Emmanuelle Le Moigne, Cécile Tromeur, Pierre Delorme, François Goffinet, Geneviève Plu Bureau, Julie Blanc, Florence Bretelle, Cécile Chau, Raoul Desbrière, Roger Rosario, Alexandra Benachi, Alexandre J. Vivanti, Laurent Mandelbrot, Edith Peynaud-Debayle, Denis Gallot, Jeannot Schmidt, Caroline Menez, Gilles Pernod, Aude Bourtembourg-Matras, Astrid Eckman, Vincent Grobost, Marc Ruivard, Emilie Gauchotte, Catherine Zuily, Aurélie Brossard, Fabrice Pierre, Franck Perrotin, Eve Mousty, Damien Laneelle, Holy Bezanahary, Chloé Schweizer, Jean-Benoît Brest, Matthieu Muller, Antoine Elias, Anne Coustel, Fabienne Comte, Gaël Beucher, Emile Ferrari, Magali Hilmi Le Roux, Frantz Bousquet, Fionnuala Ní Áinle, Brian Cleary, Jennifer Donnelly, Audrey O'Gorman, Peter MacMahon, Lucy Murphy, Alma O'Reilly, Bridgette Byrne, Kevin Ryan, Denis J. O'Keeffe, Michael Watts, Ingrid M. Bistervels, Suzanne M. Bleker, Nick van Es, Wessel Ganzevoort, Abby E. Geerlings, Saskia Middeldorp, Hanke M.G. Wiegers, Bettina Samren, Matthieu Y. van der Vlist, Sabina de Weerd, Peter E. Westerweel, Leonie de Jong-Speksnijder, Maria Simone Lunshof, Dimitri N.M. Papatsonis, Jantien Visser, Joost J. Zwart, Lucet F. van der Voet, Kim Kamphorst, Jeroen Eikenboom, Menno V. Huisman, Marieke Sueters, Karin de Boer, Marcel M.C. Hovens, Lia D.E. Wijnberger, Thomas van Bemmel, Elise S. Eerenberg, Eline S. van den Akker, Jiska M. de Haan –Jebbink, Paula F. Ypma, Ingrid Gaugler, Robbert J.P. Rijnders, Mireille N. Bekker, Maartje de Reus, Marjon A. de Boer, Johanna I.P. de Vries, Pieter-Kees de Groot, Karlijn C. Vollebregt, Gunilla Kleiverda, Marije ten Wolde, Wieteke Heidema, Mallory Woiski, Josje Langenveld, Maartje Zelis, Maureen T.M. Franssen, David P. van der Ham, Leonard P. Morssink, Brenda Hermsen, Marieke J.H.A. Kruip, Inneke Krabbendam, Claudia A. van Meir, Judith van Laar, Wim J. van Wijngaarden, Laura M. Faber, Saskia Kuipers, Hanneke van der Straaten, Nico Schuitemaker, Henk A. Bremer, Daniela Schippers, Tamara Verhagen, Annemarieke Koops, Monique A. Hertzberg, Anne F. Jacobsen, Roman G. Shmakov, Maria T. deSancho

**Affiliations:** 1 Institut National dela Santé et de la Recherche Medicale, Clinical Investigation Centre-1408, Centre Hospitalier Universitaire de St-Etienne, Saint-Etienne, France; 2 Amsterdam Universtity Medical Centre location University of Amsterdam, Department of Vascular Medicine, Amsterdam, the Netherlands; 3 Northwest Clinics, Department of Internal Medicine, Alkmaar, the Netherlands; 4 French Clinical Research Infrastructure Network INvestigation Network On Venous Thromboembolism Network, Saint-Etienne, France; 5 Spaarne Gasthuis, Department of Obstetrics and Gynaecology, Haarlem, Netherlands; 6 Department of Hematology, Rotunda Hospital and Mater Misericordiae University Hospital, Dublin, Ireland; 7 Irish Network for Venous Thromboembolism Research, Dublin, Ireland; 8 School of Medicine, University College Dublin, Dublin, Ireland; 9 Department of Cardiovascular Sciences, Vascular Medicine and Haemostasis, Katholieke Universiteit Leuven, Leuven, Belgium; 10 Department of Obstetrics and Gynaecology, Oslo University Hospital, Oslo, Norway; 11 Faculty of Medicine, University of Oslo, Hospital, Oslo, Norway; 12 Department of Clinical Biochemistry, Aalborg University Hospital, Aalborg, Denmark; 13 Department of Clinical Epidemiology, Aarhus University Hospital, Aarhus, Denmark; 14 Department of Medicine, Division of Hematology, The Ottawa Hospital, Ottawa, Canada; 15 Department of Medicine, McGill University, Montreal, Canada; 16 Division of Hematology-Oncology, Department of Medicine, New York Presbyterian Hospital, Weill Cornell Medicine, New York, USA; 17 Moscow Regional Scientific Research Institute of Obstetrics and Gynecology named after V.I. Krasnopolsky, Ministry of Healthcare of the Russian Federation, Moscow, Russia; 18 Radboud University Medical Center, Department of Internal Medicine, Nijmegen, the Netherlands; 19 Leiden University Medical Center, Department of Clinical Epidemiology, Leiden, the Netherlands; 20 Depatment of Gynécologie Obstétrique, Centre Hospitalier Universitaire de St-Etienne, Saint-Etienne, France; 21 Department of Internal Medicine & Radboud Institute of Health Sciences, Radboud University Medical Center, Nijmegen, the Netherlands

**Keywords:** clinical prediction rules, postpartum, pregnancy, pulmonary embolism

## Abstract

**Background:**

The value of pretest clinical probability scores in the diagnosis of pulmonary embolism (PE) during pregnancy and postpartum is unknown in women with a history of venous thromboembolism (VTE).

**Objectives:**

We evaluate the modified Wells, revised Geneva, and pregnancy-adapted Geneva (PAG) scores for the diagnosis of PE during pregnancy and the postpartum period in women with a history of VTE.

**Methods:**

Data from a multicenter randomized trial (Highlow) including 1110 pregnant women with a history of VTE and treated with either weight-adjusted intermediate-dose or fixed low-dose low-molecular-weight heparin subcutaneously once daily until 6 weeks postpartum were used. The modified Wells, revised Geneva, and PAG scores were calculated retrospectively in all women with a clinical suspicion of PE, and their discriminative capacity was assessed. Receiver operating characteristic (ROC) curve analysis was performed for quantitative variables and the optimal threshold defined.

**Results:**

There were 102 suspected cases of PE, of which 12 were confirmed events. During pregnancy, the ROC curves showed an area under the curve of 0.68, 0.33, and 0.36 for the Wells, Geneva, and PAG scores, respectively. During postpartum, the ROC curves showed an area under the curve of 0.75, 0.55, and 0.52 for the Wells, Geneva, and PAG scores, respectively.

**Conclusion:**

The 3 pretest clinical scores have modest discriminatory power, during both the antepartum and the postpartum period, to classify patients into 3 categories of pretest clinical probability. Further work is required to develop clinical-decision tools to exclude imaging in pregnant women with prior VTE with suspected PE in pregnancy.

## Essentials

• PE in pregnant women with a history of thromboembolism is a difficult diagnosis.
• We retrospectively evaluated predictive clinical scores in 102 pregnant women in such a situation.
• The 3 pretest clinical scores evaluated have modest discriminatory power.
• Further work is required to develop clinical-decision tools to exclude imaging in such situation.

## 1 Introduction

Pulmonary embolism (PE) is one of the leading causes of maternal death in developed countries accounting for 15% to 20% of all deaths [[1], [2], [3]]. Women with a personal history of venous thromboembolism (VTE) have a 2% to 10% absolute risk of developing recurrent VTE during a subsequent pregnancy in the absence of pharmacological thromboprophylaxis, with an odds ratio of 24.8 (95% CI, 17.1-36.0) compared with pregnant women without a history of VTE [[3], [4], [5], [6]]. Hence, for pregnant women with a history of VTE not using long-term anticoagulation, guidelines recommend postpartum thromboprophylaxis with subcutaneous low-molecular-weight heparin (LMWH) for all women and antepartum thromboprophylaxis for those at moderate or high risk of recurrent VTE [[7], [8], [9], [10]].

The diagnosis of PE in pregnant women is a challenge for clinicians because many signs or symptoms, such as dyspnea or tachycardia, which are suggestive of PE, are often related to physiological changes during pregnancy [11,12]. The diagnosis of PE is also difficult in the postpartum period as similar rates of dyspnea, chest pain, palpitations, and syncope have been observed in women with and without PE [13]. Despite an estimated low long-term risk for the mother and the fetus, doctors are reluctant to use radiating imaging in pregnant women in absence of well validated clinical pretest probability. Two prospective management outcome studies have evaluated diagnostic strategies in the specific setting of pregnant women with suspected PE. Although not previously validated in a pregnant population, 2 pretest assessment tools, the revised Geneva score [14] and the YEARS criteria, in which 3 items of the original Wells rule are combined with a D-dimer threshold [15], were used for this purpose. These 2 studies provide evidence to support radiation avoidance in 14.2% and 39% of women, respectively. However, in addition to some limitations including the use of a D-dimer cutoff not adapted to pregnancy [14] and the use of a subjective variable (PE as the most likely diagnosis) [15], imaging remains the only diagnostic option for most women. More recently, a pregnancy-adapted Geneva (PAG) score [16], which removes active malignancy and age >65 years from the initial score, was retrospectively evaluated using the Artemis data [14]. This score seems to display a better discriminate power to identify patients with PE but has not been tested in a prospective outcome study. Finally, a systematic review including 893 pregnant women, with <8% with a history of VTE, reported that the modified Wells rule (not validated in pregnant women) with fixed and adapted D-dimer threshold and the YEARS algorithm could safely rule out acute PE (failure rate, 0.37%-1.4%) [17].

The recent randomized Highlow trial [18], which compared the safety and efficacy of low and intermediate doses of LMWH during pregnancy and the postpartum period, enrolled 1110 women with a history of objectively confirmed VTE. The primary efficacy outcome was symptomatic, objectively confirmed VTE. Given the large number of pregnant women enrolled in this study who are at high risk of developing PE, this study provides a unique opportunity to evaluate the modified Wells, revised Geneva, and PAG scores for the diagnosis of PE during pregnancy and the postpartum period.

## 2 Methods

### 2.1 Patients

Between April 24, 2013, and October 31, 2020, 1110 pregnant women were randomly assigned to weight-adjusted intermediate-dose (*n* = 555) or fixed low-dose (*n* = 555) LMWH (intent-to-treat population). All these women presented with a history of objectively confirmed VTE and with a gestational age of 14 weeks or less (inclusion criteria) [18]. Eligible women were randomly assigned to weight-adjusted intermediate-dose or fixed-dose LMWH daily. LMWH was continued until 6 weeks postpartum, even if a pregnancy ended in miscarriage, abortion, or stillbirth. All women enrolled in Highlow study who presented with a symptomatic suspicion of PE at any time between randomization and 3 months postpartum were included.

### 2.2 Diagnosis of PE

PE was present if it was objectively confirmed by computed tomography pulmonary angiography, or ventilation/perfusion scan, or pulmonary angiography or if deep venous thrombosis was confirmed by lower limb venous compression ultrasonography and confirmed by the independent central adjudication committee. The diagnosis of PE was excluded in all women with negative imaging results, with no YEARS criteria and D-dimer of < 1000 ng/mL, with 1 of the 3 criteria and D-dimer of < 500 ng/mL, or with a 3-month follow-up without new thrombotic event or change in LMWH dose.

### 2.3 Clinical pretest probability of PE

Data for each woman included in Highlow trial who presented with a PE suspicion were recorded locally by the attending physician, using a case report form. Data quality was regularly monitored to detect implausible, incomplete, or inconsistent data. In addition, submitted data were compared with source medical records during periodic site monitoring visits. Using the Highlow database, the modified Wells score [19], revised Geneva score [20] and PAG score [16] were calculated retrospectively by 2 independent physicians in all women with a clinical suspicion of PE. Disagreements were resolved by consensus.

### 2.4 Statistical analysis

The objective was to evaluate the discriminative capacity of the modified Wells, revised Geneva, and PAG scores. The discrimination ability of the scores was assessed based on a receiver operating characteristic (ROC) curve. Analysis was performed using easyROC website (http://biosoft.erciyes.edu.tr/app/easyROC/) [21].

## 3 Results

Of the 1110 women included, a total of 102 suspected cases of PE were reported, mainly after the first trimester of pregnancy (Table 1). These suspected cases of PE occurred in 99 women (9%), with 2 women having 2 consecutive suspected cases of PE, 2 women in the third trimester and then in the postpartum period, and 1 woman in the first and third trimesters. A total of 78 and 24 suspected cases of PE occurred in the antenatal and postnatal periods, respectively.

**Table 1:** Pregnancy and postpartum period of pulmonary embolism suspicion.

Period of pregnancy	No. of PE suspicion (%)	Confirmed PE (%)	PE	Percentage of confirmed/ruled out PE
	102	12	90	11.8/88.2
Antenatal period	78	2	76	2.6/97.4^a^
First trimester	11 (11)	0	11 (12)	0/100
Second trimester	41 (40)	0	41 (46)	0/100
Third trimester	26 (25)	2 (17)	24 (27)	7.7/92.3
Postpartum	24 (24)	10 (83)	14 (16)	41.7/58.3^a^

PE, pulmonary embolism.

a Between antenatal and postpartum period (*P* < .00001).

### 3.1 Antepartum period

**Figure 1:**
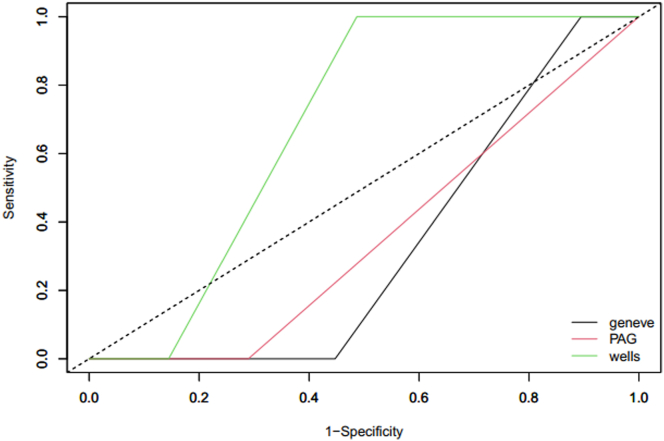
Receiver operating characteristic curves comparison of Wells score (green), Geneva score (black), and pregnancy-adapted Geneva (PAG) score (red) for pulmonary embolism suspicions during antepartum period.

A total of 78 suspected cases occurred during the antenatal period. The clinical characteristics of these suspected cases are shown in Table 2. During this period, in addition to a 3-month follow-up without new events, PE was excluded in 54 suspected cases (67%) by lung imaging and in 24 suspected cases (32%) by YEARS and D-dimer criteria. In a suspected episode of PE, the diagnosis of PE was ruled out by both negative-result compression ultrasound and negative-result transthoracic echocardiography in a woman treated for hypothyroidism who presented with dyspnea on exertion only in the first trimester of pregnancy. This woman had no suspected PE during her 3-month follow-up. Two women presented with confirmed PE occurring in the third trimester, giving a PE incidence of 2.6% among the 78 suspected cases. The distribution of suspected PE and the corresponding incidence of PE according to the clinical risk scores are shown in Table 3. Irrespective of the clinical score considered, no woman with confirmed PE had a low clinical probability score, but the distribution of women with a low probability score was very small using the Geneva and PAG scores (0% and 10.3%, respectively). An intermediate probability score was present in two-thirds of women and more than 8 of 10 women using the Wells, Geneva, and PAG scores respectively. The ROC curves showed an area under the curve of 0.68 (95% CI, 0.60-0.77), 0.33 (95% CI, 0.25-0.45), and 0.36 (95% CI, 0.30-0.41) for the Wells, Geneva, and PAG scores, respectively (Figure 1).

**Table 2:** Characteristics of pulmonary embolism suspicion during the antepartum period.

Characteristic	Confirmed PE (*n* = 2), *n* (%)^a^	PE ruled out (*n* =76), *n* (%)^a^
Age (y), mean ± SD	—	—
Trimester of pregnancy		
First	0	11 (14)
Second	0	41 (54)
Third	2 (100)	24 (32)
BMI at inclusion (kg/m^2^)	—	—
Primigravidity	—	—
Nulliparity	—	—
Personal previous PE	2 (100)	42 (55)
During previous pregnancy	2	13
During previous postpartum	0	7
Personal previous DVT	0	34 (45)
Low-dose LWMH	2 (100)	42 (55)
Intermediate-dose LMWH	0	34 (45)
Duration of PE symptoms		
<24 h	1 (50)	30 (39)
Between 24 h and 1 wk	1 (50)	21 (28)
>1 wk	0	14 (18)
Unknown	0	11 (14)
Chest pain	2 (100)	43 (57)
Dyspnea	2 (100)	58 (76)
Syncope/lipothymia	1 (50)	7 (9)
Hemoptysis	0	0
Clinical signs or symptoms of DVT	0	8 (11)
Heart rate (bpm)		
>100	0	20 (26)
>94^b^	1 (50)	29 (38)
PE as the most likely diagnosis	2 (100)	34 (45)
CTPA	1 (50)	37 (48)
V/Q scan	0	13 (17)
Both CTPA and V/Q scan	1 (50)	1 (1)
No lung imaging	0	25 (33)
Legs ultrasonography	0	53 (70)
D-dimer evaluation		
No years criteria and D-dimer < 1000 ng/mL	0	17 (22)^c^
1 or more years criteria and D-dimer < 500 ng/mL	0	7 (9)^d^

CT, computed tomography; CTPA, computed tomographic pulmonary angiography; LMWH, low-molecular-weight heparin; V/Q, ventilation/perfusion.

a No pregnant women presented with either an active cancer or a surgery within previous month.

b In 10 additional cases, the heart rate was reported as lower than 100 bpm without the knowledge of the exact rate (lower or upper 94 bpm).

c In 3 cases, a CT scan was performed and showed negative results.

d In 1 case, a CT scan was performed and showed negative results.

**Table 3:** Pulmonary embolism suspicions’ distribution and corresponding incidence of pulmonary embolism according to the clinical risk scores during antepartum period.

Clinical score	Category of clinical probability		
	Low	Intermediate	High
Modified Wells score	<2	2-6	>6
No. of suspicions	28	48	2
Distribution (%)	35.9	61.5	2.6
Confirmed PE (*n*)	0	2	0
Incidence of PE (%; 95% CI)	0 (NA)	4.2 (0.0-9.8)	0 (NA)
Revised Geneva score	≤3	4-10	>10
No. of suspicions	8	68^a^	2
Distribution (%)	10.3	87.2	2.6
Confirmed PE (*n*)	0	2	0
Incidence of PE (%; 95% CI)	0 (NA)	2.9 (0.0-7.0)	0 (NA)
PAG score	<2	2-6	≥7
No. of suspicions	0	66	12
Distribution (%)	0	84.6	15.4
Confirmed PE (*n*)	0	2	0
Incidence of PE (%; 95% CI)	0 (NA)	3 (0.0-7.2)	0 (NA)

PAG, pregnancy-adapted Geneva; PE, pulmonary embolism.

a In absence of the exact number of the heart rate, the score was 6 or 8 for 10 patients.

### 3.2 Postpartum period

A total of 24 suspected PE cases occurred in the postpartum period. The clinical characteristics of these 24 suspected cases are shown in Table 4. During this period, in addition to 3 months of follow-up without a new event, PE was excluded in 11 suspected cases (79%) by negative-result pulmonary imaging and in 2 suspected cases (14%) by D-dimer of <500 ng/mL. In 1 additional case, PE was suspected 2 weeks after spontaneous delivery without complications. In this case, PE was excluded due to low clinical probability (Wells score of 1.5) and a diagnosis of anemia, explaining the complaint of dyspnea on exertion. This woman did not present with suspected PE during her 3-month follow-up. Ten women presented with confirmed PE during the postpartum period, giving a PE incidence of 42% among PE suspects.

**Table 4:** Characteristics of pulmonary embolism suspicion during the postpartum period.

Characteristics	Confirmed PE (*n* = 10), *n* (%)	PE ruled out (*n* = 14), *n* (%)
Age (y), mean ± SD	—	—
Personal previous PE	7 (70)	11 (79)
Personal previous DVT	6 (60)	7 (50)
Nulliparity	—	—
Low-dose LMWH	7 (70)	7 (50)
Intermediate-dose LMWH	3 (30)	7 (50)
Cesarean section	3 (30)	6 (43)^a^
Emergency cesarean section	3 (100)	2 (33)
Time after delivery		
≤1 wk	4 (40)	9 (64)
<1-6 wk	4 (40)	4 (29)
˃6 wk^b^	2 (20)	1 (7)^c^
Major bleeding or clinically relevant nonmajor bleeding		
Antepartum	0	0
Early postpartum	0	4 (29)
Late postpartum	0	0
Thorax pain	9 (90)	10 (71)
Dyspnea	7 (70)	10 (71)
Syncope/lipothymia	2 (20)	1 (7)
Hemoptysis	0	0
Clinical signs or symptoms of DVT	2 (20)	2 (33)
Heart rate (bpm)		
>100	4 (40)	3 (21)
>94^d^	5 (50)	6 (43)
PE as the most likely diagnosis	10 (100)	6 (43)
CTPA	10 (100)	11 (79)
V/Q scan	0	0
Both CTPA and V/Q scan	0	0
No lung imaging	0	3 (21)
Legs ultrasonography	3 (30)	1 (7)
D-dimer < 500 ng/mL	0	2 (14)

bpm, beats per minute; CTPA, computed tomography pulmonary angiography; DVT, deep vein thrombosis; LMWH, low-molecular-weight heparin; PE, pulmonary embolism; V/Q, ventilation/perfusion.

a In 1 case, the cesarean section was planned because of previous cesarean delivery, and in 3 cases, the indication was not reported.

b In Highlow study protocol, venous thromboembolism occurrence was evaluated from randomization up to 6 wk postpartum.

c In 1 case, PE suspicion occurred 44 d after miscarriage.

d In 5 additional cases, the heart rate was reported as lower than 100 bpm without the knowledge of the exact rate (lower or upper 94 bpm).

**Figure 2:**
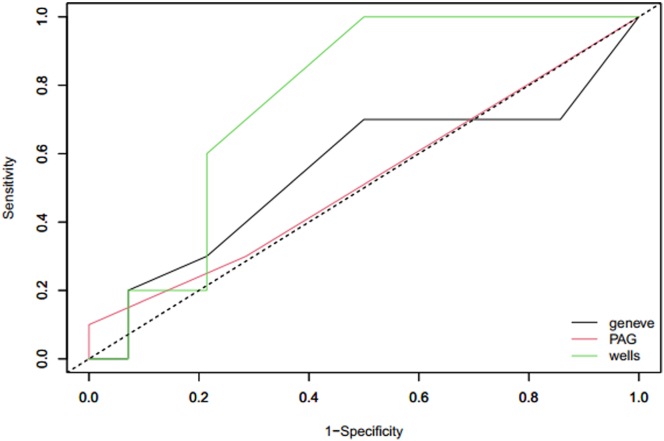
Receiver operating characteristic curves comparison of Wells Score (green), Geneva score (black), and pregnancy-adapted Geneva (PAG) score (red) for pulmonary embolism suspicions during postpartum period.

The distribution of suspected PE and the corresponding incidence of PE according to the clinical risk scores are shown in Table 5. No PE was observed in women with a low probability score using the Wells and PAG scores, but the distribution of such women was low (16.7% and 0%, respectively). PE was diagnosed in 60% of women with a low probability score using the Geneva score. PE was diagnosed in one-third to one-half of women with an intermediate probability score using the Geneva, PAG, and Wells scores. The ROC curves showed an area under the curve of 0.75 (95% CI, 0.56-0.95), 0.55 (95% CI, 0.30-0.80), and 0.52 (95% CI, 0.32-0.72) for the Wells, Geneva, and PAG scores, respectively (Figure 2).

**Table 5:** PE suspicions’ distribution and corresponding incidence of pulmonary embolism according to the clinical risk scores during postpartum.

Clinical score	Category of risk		
	Category of clinical probability		
	Low	Intermediate	High
Modified Wells score	<2	2-6	>6
No. of suspicions	4	15	5
Distribution (%)	16.7	62.5	20.8
Confirmed PE (*n*)	0	8	2
Incidence of PE (%; 95% CI)	0 (NA)	53.3 (28.1-78.6)	40 (0.0-82.9)
Revised Geneva score	≤3	4-10	>10
No. of suspicions	5	16^a^	3
Distribution (%)	20.8	66.7	12.5
Confirmed PE (*n*)	3	5	2
Incidence of PE (%; 95% CI)	60 (17.1-100.0)	31.3 (8.5-54.0)	66.7 (13.3-100.0)
PAG score	<2	2-6	≥7
No. of suspicions	0	20	4
Distribution (%)	0	83.3%	16.7%
Confirmed PE (*n*)	0	8	2
Incidence of PE (%; 95% CI)	0 (NA)	40 (18.5-61.5)	50 (1.0-99.0)

PAG, pregnancy-adapted Geneva; PE, pulmonary embolism.

a In absence of the exact number of the heart rate, the score was 6 or 8 for 4 patients.

## 4 Discussion

This work presents the evaluation of 3 clinical-decision rules based on the Wells, Geneva, and PAG stand-alone scores without D-dimer for the assessment of clinical pretest probability in women with a personal history of VTE during pregnancy and the postpartum period and with suspected PE. The high-risk population of pregnant women with prior VTE with suspected PE has been very poorly studied, representing only 7.3% (29/395) [14] and 6% (30/498) [15] of the women included in the 2 recent studies evaluating a PE diagnostic strategy in pregnant women. In this study, despite their high thrombosis risk, the incidence of confirmed PE during pregnancy was low, 0.2% (2/1110), probably due to the treatment with low or intermediate doses of LMWH initiated from gestational age of ≤14 weeks until 6 weeks postpartum. During the postpartum period, the incidence of confirmed PE was 0.9% (10/1110), which is lower than previously reported rate (6.5%) in a study of women with a history of VTE (138 pregnancies) [5], where >50% of the pregnancies were without LMWH prophylaxis.

Our results show that the 3 pretest clinical scores have modest discriminatory power, for both the antepartum and the postpartum periods, to classify patients into 3 categories of pretest clinical probability. Indeed, during pregnancy, even if the incidence of PE was 0% in women classified as having a low probability score by each of the 3 scores, the distribution of patients for this probability was at most 36% for the Wells score and <11% for the Geneva and PAG scores. There are several reasons for this observation. For the Wells score, which has the better discriminatory power, some of the 7 criteria, such as malignancy, surgery, or immobilization in the previous 4 months, are very rarely reported by pregnant women at the time of their suspicion of PE. In fact, the prevalence of malignancy, surgery in the previous 4 weeks, immobilization (for >3 days), and hemoptysis were 0%, 1%, 6.2%, and 7.7% in the ARTEMIS study [15] and 0%, 1%, 8.6%, and 3.5% in the study reported by Righini et al [14]. Thus, these reasons could explain the greater number of patients classified as low clinical probability using the Wells score. For the PAG score, the presence of a history of VTE counts for 3 points, which immediately excludes the entire population evaluated as having a low clinical probability. For the Geneva score, both a history of PE and a heart rate >75 beats per minute (often observed during pregnancy) count for 3 points making the score sufficient to be classified as least an intermediate probability for PE. In addition, for any of the 3 clinical probability scores, we did not observe a high discriminant power allowing to classify patients in 3 categories associated with clinically meaningful increasing PE incidence. The low number of women with confirmed PE observed in our study could be an explanation. However, as most women were classified in the intermediate probability category, the variables used for these scores do not seem to be adequate to pregnant women with high thrombotic risk despite LMWH prophylaxis. Based on our study, it is necessary to identify better characteristics that can discriminate PE in pregnant women at high thrombotic risk. Some new features such as the use of LMWH prophylaxis, the provoked or nonprovoked nature of the incident VTE, and the presence of severe thrombophilia may be taken into account.

In the postpartum period, the value of the 3 clinical pretest probability scores appears to be of little use. In fact, the patient distributions for this probability were at most 17% for the Wells score and 0% for the PAG score, even though the incidence of PE was 0% in women classified as having a low probability score using the Wells and PAG scores. In addition, in the 5 women who were classified as having a low probability score according to the Geneva score, a PE incidence of 60% was observed. In this score, 3 of 8 items, such as age >65 years, cancer and surgery (under general anesthesia), or having fractured a lower limb in the past month, are not relevant in this population. Furthermore, little direct data supported the use of 2 other items: unilateral leg pain and pain on palpation, and unilateral edema (observed in <17% in our study). Therefore, with 5 of 8 items rarely observed in the postpartum period, it is not surprising to find confirmed cases of PE in women classified as low probability risk. In this respect, the PAG score, which removed some of these items and introduced the threshold of >110 bpm, showed better results. However, most women (83%) with confirmed PE were classified as being at intermediate risk. Again, there is a need to better identify features associated with PE in postpartum women at high risk of VTE. For example, the Geneva score may have incorrectly excluded PE in 3 women because of the low clinical probability. All 3 women with a confirmed PE despite a low probability of risk using the Geneva score had a history of VTE related to hormonal contraceptives, which is not considered by the different scores. Other characteristics such as intrauterine growth restriction, preterm birth and pre-eclampsia, cesarean section, and postpartum hemorrhage, which are known to increase the risk of VTE [22], should also be entertained.

The main limitation of this work is the low number of suspected and confirmed PEs in our source data, including 78 women during antepartum period of whom only 2 presented a confirmed PE and 24 women during the postpartum period with 10 confirmed PEs. Therefore, it was not possible to identify the potentially significant variables associated with the diagnosis of PE. However, this limitation is inherent to this selected population of women at high thrombotic risk, whereas studies on PE diagnosis are challenging to conduct in such a population. It took 8 years and the mobilization of 70 centers in 9 countries to enroll 1110 pregnant women in the Highlow study [18]. The lack of data on ethnicity/race may also represent a limitation of our study. However, there are no data in the literature to suggest a difference in terms of incidence of venous thromboembolic disease or symptoms that may suggest this diagnosis in pregnant women according to ethnicity/race. Furthermore, given the rarity of this diagnosis in pregnant women with a history of VTE, it is unlikely that this will ever be demonstrated.

In women at high risk of thromboembolism, the assessment of pretest probability in pregnant and postpartum women with suspected PE is particularly challenging. The strength of the history of VTE at the time of suspicion seems to take precedence over the clinical elements of the suspicion [5,23,24]. This may explain why, despite the high frequency of an alternative diagnosis of PE (a subjective predictor) including symptoms related to physiological changes in pregnancy (50% of cases in our study), a computed tomography pulmonary angiography is almost always performed. However, as the incidence of PE in women at high thrombotic risk receiving LMWH prophylaxis is low, there is a need to find alternative strategies to minimize the proportion of pregnant women who need chest radiation imaging [23,24]. The relevance of 2 current diagnostic algorithms [14,15] including the revised Geneva score, the YEARS criteria, and D-dimer test for the diagnostic of PE in pregnant women with a history of VTE and on LMWH prophylaxis is controversial as very few of such women were included in these studies and the potential effect of LMWH on D-dimer levels during pregnancy is unknown.

In conclusion, the Wells, Geneva, and PAG clinical scores have modest discriminatory power in women with prior VTE who present with suspected PE in pregnancy during both the antepartum and the postpartum periods. Further work is required to develop clinical-decision tools to exclude radiation imaging in pregnant women with prior VTE with suspected PE in pregnancy.

## Appendix

**Members of Highlow Investigators:** Barbara Debaveye, Kathelijne Peerlinck, Caroline P. Martens,Kristine Vanheule, Thomas Vanassche, Peter Verhamme: University Hospital Leuven, Leuven, Belgium. Marc A. Rodger, Alan Karovitch: the Ottawa Hospital, Ottawa, Canada. Anette Tarp Hansen, Aiste Kloster: Aalborg University Hospital, Aalborg, Denmark. Jens Fuglsang: Aarhus University Hospital, Aarhus, Denmark. Andrea Buchmüller, Céline Chauleur, Cécile Duvillard, Nathalie Moulin, Tiphaine Raia-Barjat, Thomas Corsini, Suzanne Lima: Centre Hopitalo-Universitaire, Saint Etienne, France. Luc Bressollette, Francis Couturaud, Karine Lacut (deceased), Emmanuelle Le Moigne, Cécile Tromeur: Centre Hospitalier Universitaire, Brest, France. Pierre Delorme, François Goffinet, Geneviève Plu Bureau: Assistance Publique Hôpitaux de Paris Port Royal, Paris Cité, France. Julie Blanc, Florence Bretelle, Cécile Chau: Hôpital Nord, Marseille, France. Raoul Desbrière, Roger Rosario: Hôpital Saint Joseph, Marseille, France. Alexandra Benachi, Alexandre J. Vivanti: Assistance Publique Hôpitaux de Paris, Antoine Béclere, Clamart, France. Laurent Mandelbrot, Edith Peynaud-Debayle: Assistance Publique Hôpitaux de Paris Louis-Mourier, Colombes, France. Denis Gallot, Jeannot Schmidt: Centre Hospitalier Universitaire de Clermont-Ferrand, Service d'aide Médicale Urgente, Clermont-Ferrand, France. Caroline Menez, Gilles Pernod: Centre Hospitalier Universitaire de Grenoble, La Tronche, France. Aude Bourtembourg-Matras, Astrid Eckman: Centre Hospitalier Universitaire de Besançon, Besançon, France. Vincent Grobost, Marc Ruivard: 15 Centre Hospitalier Universitaire de Clermont-Ferrand, Service de Médecine Vasculaire Clermont-Ferrand, France. Emilie Gauchotte, Catherine Zuily-Lamy: Centre Hospitalier Universitaire de Nancy, Nancy, France. Aurélie Brossard, Fabrice Pierre: Centre Hospitalier Universitaire de Poitiers, Poitiers, France. Franck Perrotin: Centre Hospitalier Universitaire de Tours, Tours, France. Eve Mousty: Centre Hospitalier Universitaire de Nîmes, Nîmes France. Damien Laneelle: Centre Hospitalier Universitaire de Caen, Caen, France. Holy Bezanahary: Centre Hospitalier Universitaire de Limoges, Limoges, France. Chloé Schweizer: Centre Hospitalier Universitaire de La Réunion, Saint Pierre, France. Jean-Benoît Brest, Matthieu Muller: Centre Hospitalier des Pays de Morlaix, Morlaix, France. Antoine Elias: Centre Hospitalier Intercommunal de Toulon, Toulon, France. Anne Coustel: Centre Hospitalier Universitaire de Bordeaux, Bordeaux, France. Fabienne Comte: Centre Hospitalier de Roanne, Roanne, France. Gaël Beucher: Centre Hospitalier Universitaire de Caen Normandie, Caen, France. Emile Ferrari, Magali Hilmi Le Roux: Centre Hospitalier Universitaire de Nice, Nice, France. Frantz Bousquet: Polyclinique Sainte Thérèse de Sète, Sète, France. Fionnuala Ní Áinle, Brian Cleary, Jennifer Donnelly, Audrey O'Gorman, Peter MacMahon, Lucy Murphy, Alma O'Reilly: Rotunda Hospital, Dublin, Ireland. Bridgette Byrne, Kevin Ryan: The Coombe Women & Infants University Hospital, Dublin, Ireland. Denis J. O'Keeffe, Michael Watts: University Hospital Limerick, Limerick, Ireland. Ingrid M. Bistervels, Suzanne M. Bleker, Nick van Es, Wessel Ganzevoort, Abby E. Geerlings, Saskia Middeldorp, Hanke M.G. Wiegers: Amsterdam UMC, location University of Amsterdam, Amsterdam, the Netherlands. Bettina Samren, Matthieu Y. van der Vlist, Sabina de Weerd, Peter E. Westerweel: Albert Schweitzer Hospital, Dordrecht, the Netherlands. Leonie de Jong-Speksnijder, Maria Simone Lunshof, Dimitri N.M. Papatsonis, Jantien Visser: Amphia Hospital, Breda the Netherlands. Joost J. Zwart, Lucet F. van der Voet, Kim Kamphorst: Deventer Hospital, Deventer, the Netherlands. Jeroen Eikenboom, Menno V. Huisman, Marieke Sueters: Leiden University Medical Center, Leiden, the Netherlands. Karin de Boer, Marcel M.C. Hovens,Lia D.E. Wijnberger: Rijnstate Hospital, Arnhem, the Netherlands. Thomas van Bemmel, Elise S. Eerenberg: Gelre Hospital, Apeldoorn, the Netherlands. Eline S. van den Akker, Jiska M. de Haan –Jebbink: OLVG Oost Hospital, Amsterdam, the Netherlands. Paula F. Ypma: Haga Hospital, The Hague, the Netherlands. Ingrid Gaugler, Robbert J.P. Rijnders: Jeroen Bosch Hospital, ‘s-Hertogenbosch, the Netherlands. Mireille N. Bekker, Maartje de Reus: University Medical Center Utrecht, Utrecht, the Netherlands. Marjon A. de Boer, Johanna I.P. de Vries: Amsterdam UMC, location Vumc, Amsterdam, the Netherlands. Pieter-Kees de Groot, Karlijn C. Vollebregt: Spaarne Gasthuis, Haarlem the Netherlands. Gunilla Kleiverda, Marije ten Wolde: Flevo Hospital, Almere, the Netherlands. Wieteke Heidema, Mallory Woiski: Radboud University Medical Center, Nijmegen, the Netherlands. Josje Langenveld, Maartje Zelis: Zuyderland Medical Centre, Heerlen, the Netherlands. Maureen T.M. Franssen: University Medical Center Groningen, Groningen, the Netherlands. David P. van der Ham: Martini Hospital, Groningen, the Netherlands. Leonard P. Morssink: Medical Center, Leeuwarden, Leeuwarden, the Netherlands. Brenda Hermsen: OLVG West Hospital, Amsterdam, the Netherlands. Marieke J.H.A. Kruip: Erasmus Medical Center, Rotterdam, the Netherlands. Inneke Krabbendam: Hospital Gelderse Vallei, Ede, the Netherlands. Claudia A. van Meir: Groene Hart Hospital, Gouda the Netherlands. Judith van Laar: Máxima Medical Center, Veldhoven, the Netherlands. Wim J. van Wijngaarden: Medical Center Haaglanden, The Hague, the Netherlands. Laura M. Faber: Rode Kruis Hospital, Beverwijk, the Netherlands. Saskia Kuipers: Admiraal de Ruijter Hospital, Goes, the Netherlands. Hanneke van der Straaten: St Jansdal Hospital, Harderwijk, the Netherlands. Nico Schuitemaker: Diakonessen Hospital, Utrecht, the Netherlands. Henk A. Bremer: Reinier de Graaf, Gasthuis, Delft, the Netherlands. Daniela Schippers: Canisius-Wilhelmina Hospital, Nijmegen, the Netherlands. Tamara Verhagen: Slingeland Hospital, Doetichem, the Netherlands. Annemarieke Koops: Wilhelmina Hospital, Assen, the Netherlands. Monique A. Hertzberg, Anne F. Jacobsen: Oslo University Hospital, Oslo, Norway. Roman G. Shmakov: Institute of Obstetrics, National Medical Research Center for Obstetrics, Gynecology and Perinatology named after V.I. Kulakov, Moscow, Russia. Maria T. deSancho: New York Presbyterian Hospital, New York, USA.
